# Improved Synthesis of 5-Nitrohomovanillic Acid and 6-Nitrohomovanillic Acid as Probes for Metabolism Studies of Endothelium-Derived Dopamines: Identification in Human Amniotic Fluid

**DOI:** 10.3390/molecules30204096

**Published:** 2025-10-15

**Authors:** Rosa Sparaco, Pierfrancesco Cinque, Antonia Scognamiglio, Stefania Vertuccio, Giuseppe Caliendo, Ferdinando Fiorino, Angela Corvino, Elisa Magli, Elisa Perissutti, Vincenzo Santagada, Beatrice Severino, Giorgia Andreozzi, Paolo Luciano, Carmela Dell’Aversano, Alex Henrique Miller, Gilberto De Nucci, Francesco Frecentese

**Affiliations:** 1 Department of Pharmacy, University of Naples Federico II, Via D. Montesano 49, 80131 Naples, Italy; rosa.sparaco@unina.it (R.S.); pierfrancesco.cinque@unina.it (P.C.); antonia.scognamiglio@unina.it (A.S.); stefania.vertuccio@unina.it (S.V.); angela.corvino@unina.it (A.C.); perissut@unina.it (E.P.); santagad@unina.it (V.S.); bseverin@unina.it (B.S.); giorgia.andreozzi@unina.it (G.A.); pluciano@unina.it (P.L.); dellaver@unina.it (C.D.); frecente@unina.it (F.F.); 2 Department of Public Health, University of Naples Federico II, Via Pansini 5, 80131 Naples, Italy; elisa.magli@unina.it; 3 Department of Pharmacology, Faculty of Medical Sciences, State University of Campinas (UNICAMP), Campinas 13083-888, SP, Brazil; amiller@unicamp.br (A.H.M.); denucci@unicamp.br (G.D.N.); 4 Department of Pharmacology, Faculdade São Leopoldo Mandic, Campinas 13045-755, SP, Brazil

**Keywords:** 5-nitrohomovanillic acid, 6-nitrohomovanillic acid, 6-nitrodopanine, high-resolution mass spectrometry, NMR, human amniotic fluid

## Abstract

6-Nitrodopamine is an endogenous catecholamine responsible for numerous biological activities. Here, an improved method for the synthesis of both 6-nitrohomovanillic acid (6-NHVA) and its regioisomer 5-nitrohomovanillic acid (5-NHVA) is reported. The developed one-step synthetic procedures ensured the efficient preparation of the target compounds in good yields. Comprehensive structural characterization was achieved through one- and two-dimensional NMR studies and by high-resolution mass spectrometry (HR-MS/MS). The presence of both substances was identified in human amniotic fluid by LC-MS/MS.

## 1. Introduction

Homovanillic acid (HVA) is one of the main degradation products of dopamine (DA). Monoamine oxidase (MAO) catalyzes the first of a two-step reaction that leads to DA deamination to 3,4-dihydroxyphenylacetaldehyde (DOPAL). This is in turn metabolized by aldehyde dehydrogenase (ALDH) into 3,4-dihydroxyphenylacetic acid (DOPAC). Finally, O-methylation of DOPAC by catecholamine O-methyltransferase (COMT) produces HVA [1]. Alternatively, DA can be primarily O-methylated by COMT to produce 3-methoxytyramine (MT). MT is then converted into 3-methoxy-4-hydroxyacetaldehyde (MHAcDH) by MAO. Then, HVA is obtained through the conversion of MHAcDH by ALDH [2].

6-Nitrodopamine (6-ND) is the main catecholamine released from both the endothelium [3,4] and epithelium [5] of mammalian tissues. As a positive chronotropic agent, it is more potent than noradrenaline and adrenaline (>100 times), and far more potent than dopamine (~10,000 times) in the rat isolated right atrium [6]. In humans, the 6-ND basal release was identified from umbilical cord vessels [7], popliteal vessels [3], vas deferens [8], and seminal vesicles [5].

In contrast to 6-cyanodopamine and 6-bromodopamine, no circulating levels of 6-ND have been detected [9]. This is not surprising, since, as mentioned above, 6-ND is remarkably potent both in vitro [6] and in vivo [10]. Since the major metabolite of dopamine is homovanillic acid, it is possible that 6-nitrodopamine can likely be metabolized in a similar manner, giving rise to 6-nitrohomovanillic acid. Thus, the obtention of this possible metabolite should provide a useful tool for biochemical studies on the metabolism of 6-nitrodopamine.

The objective of this study was to initially develop an optimized synthetic process for obtaining 6-nitrohomovanillic acid (6-NHVA). Given that a retrospective literature review revealed that the synthesis of 5-nitrohomovanillic acid had been described for the first time in 1983 [11], and successively in 1986 [12] and 2003 [13], we decided to focus our attention on this nitro derivative of homovanillic acid, optimizing the synthetic procedure for it as well. Obtaining both nitrated derivatives of homovanillic acid allowed us to conduct comparative structural studies and thus extend the validation of an analytical method for research in human biological fluids not only to 6-NHVA, a potential metabolite of 6-ND, but also to 5-NHVA.

Homovanillic acid is the major metabolite of dopamine excreted in urine [14,15,16]. Amniotic fluid is fetal urine [17], in which dopamine, produced by human amniotic epithelial cells [18], is the major catecholamine found [19]. Since HVA has been measured/identified in this matrix [20,21], identification of the potential metabolites 6-NHVA and 5-NHVA in amniotic fluid may indicate in vivo production of both 6-ND and 5-ND (a 6-ND positional isomer), respectively.

Upon completion of the synthesis steps and setting the optimized chromatographic conditions for the separation and identification of the two novel regioisomers, a detailed structural characterization by one- and two-dimensional NMR experiments was carried out, allowing assignment of the nitro group position on the aromatic ring. Moreover, a high-resolution mass spectrometry study was conducted to describe the HRMS/MS fragmentation pattern of the synthesized molecules. Mass spectrometry results laid the foundations to develop analytical methods for the detection of these compounds in biological matrices. For completeness, NMR and HRMS/MS relevant data for the 6-ND, 6-NHVA, and 5-NHVA were compared.

## 2. Results

### 2.1. Synthesis and Chemical–Physical Characterization

The study of the metabolism of 6-ND, which has been widely studied recently as a new endogenous mediator [4], has focused our attention on the synthesis of 6-NHVA (Compound **I**).

Our study commenced with a survey of procedures already published in the literature for the preparation of the target compound.

The preparation of this metabolite has been described in only two historical references, with the most recent publication dating back to 1949 [22,23]. Both scientific articles describe multi-step synthetic methodologies that contrast with modern medicinal chemistry principles focused on green approaches and a favorable cost–benefit profile. In the latter paper, the compound was obtained starting from eugenyl acetate that was nitrated by reaction with nitric acid in acetic anhydride and deprotected with methanolic potassium hydroxide to give 6-nitroeugenol. This was re-protected on the free phenol by treatment with acetyl chloride and acetic anhydride in pyridine, and finally, the obtained intermediate, corresponding to 6-nitroeugenyl acetate, was oxidized by potassium permanganate in an acidic aqueous medium. Such procedures led to low yields of the compound of interest.

**Scheme 1:**
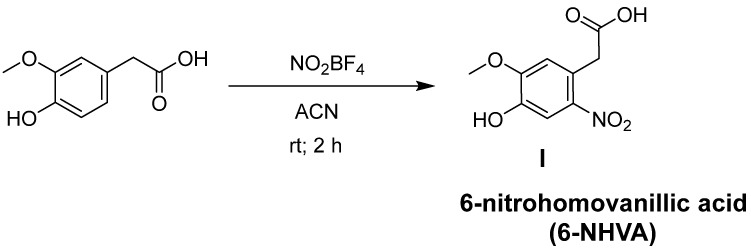
Synthetic procedure for the synthesis of 6-nitrohomovanillic acid.

Our goal was the direct nitration of homovanillic acid, and our optimized procedure was based on the use of nitronium tetrafluoroborate in anhydrous acetonitrile, as reported in Scheme 1.

The reaction gave a good yield of the desired 6-nitroregioisomer. The reaction was performed at room temperature for 2 h, and the purification, for the removal of unreacted nitronium tetrafluoroborate, was carried out by RP-HPLC.

For 5-nitrohomovanillic acid (compound **II**), as reported above in the introduction, we found that the synthesis starting from homovanillic acid had only been reported in a Beecham Pharmaceutical Group patent in 1983. The procedure consists of direct nitration of homovanillic acid with fuming nitric acid and glacial acetic acid, leading to a 55 percent yield [10]. This procedure was later reported by the same research group, without modifications, also in *The Journal of Antibiotics* in 1986 [12].

Apart from this strategy, there is only one other paper in the literature, published in 2003, for the preparation of compound **II**, but in this case, the synthetic strategy started from vanillin [13]: the compound was acetylated on the free hydroxyl group and treated with fuming nitric acid. This reaction led to the 5-nitroderivative in only 10 percent yield. Furthermore, the approach to 5-nitrohomovanillic acid in this paper required three more steps with procedures that therefore made this strategy less amenable to the preparation of this compound in high yield.

**Scheme 2:**
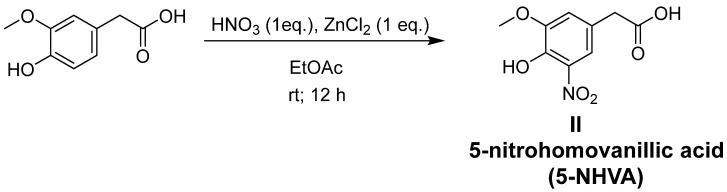
Synthetic procedure for the synthesis of 5-nitrohomovanillic acid.

In the literature, numerous nitration synthetic protocols are reported and discussed: among these, based on our expertise, we took inspiration from efficient and selective methods based on the use of nitronium tetrafluoroborate or nitric acid/zinc chloride, adopting them for our purpose. Our goal was the direct nitration of homovanillic acid, and our optimized procedure is based on the use of zinc chloride as a catalyst, as reported in Scheme 2.

The isolated compounds were analyzed by RP-HPLC, paying attention to their absolute and relative retention times to identify the optimized chromatographic conditions that allow good separation of the regioisomers in the mixture. Chromatograms are reported in the Supplementary Materials (Figures S1–S4).

The exact assignment of position 5 or 6 of the nitro group on the aromatic ring for 6-ND, 6-NHVA, and 5-NHVA was carried out by NMR studies. Indeed, the analysis of one- and two-dimensional NMR spectra enabled the structure of the compounds under investigation to be unambiguously established. ^1^H, ^13^C, HSQC, and HMBC NMR spectra were acquired. This latter experiment, through correlation analysis, allows the position of the nitro group to be definitively assigned.

**Figure 1:**
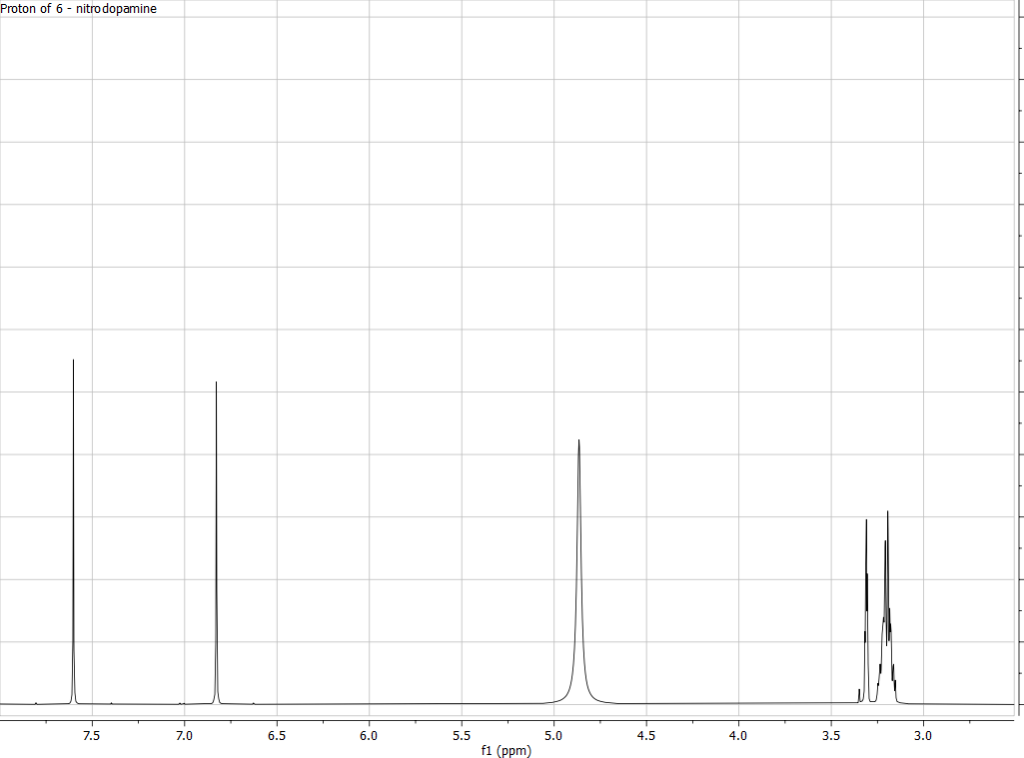
^1^H-NMR of 6-nitrodopamine in CD_3_OD.

**Figure 2:**
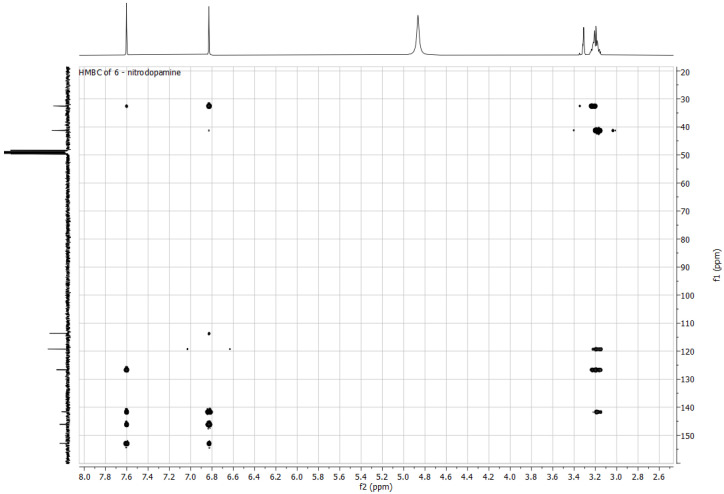
HMBC of 6-nitrodopamine in CD_3_OD.

**Figure 3:**
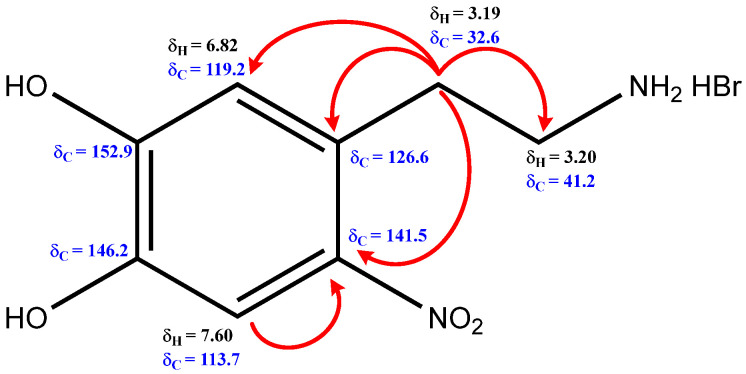
Structure assignment for 6-nitrodopamine hydrobromide.

Our NMR characterization started from 6-nitrodopamine, which is already known in the literature [3]. The proton NMR spectrum, HSQC spectrum, and the chemical structure, along with the main NMR correlations, are reported in Figure 1, Figure 2 and Figure 3, respectively.

**Figure 4:**
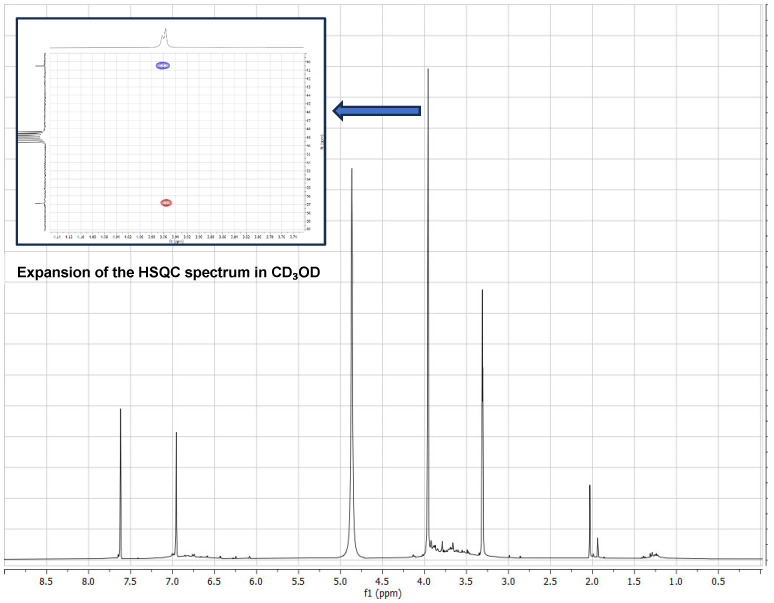
^1^H-NMR of 6-nitrohomovanillic acid in CD_3_OD.

**Figure 5:**
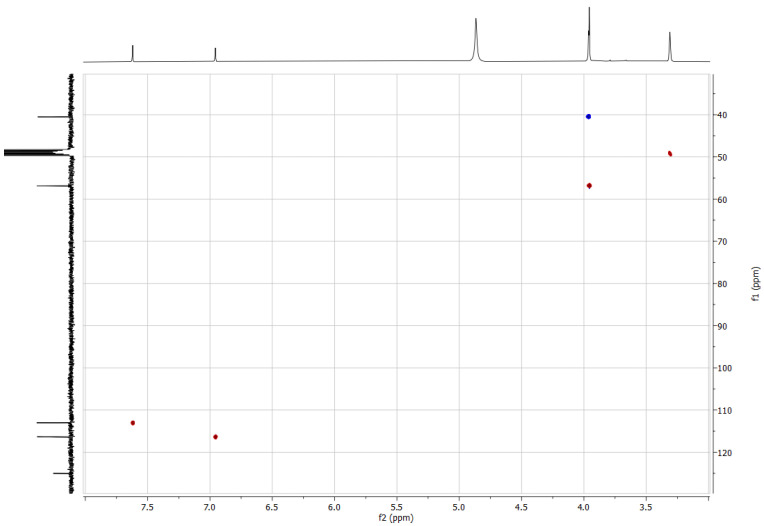
HSQC of 6-nitrohomovanillic acid in CD_3_OD.

**Figure 6:**
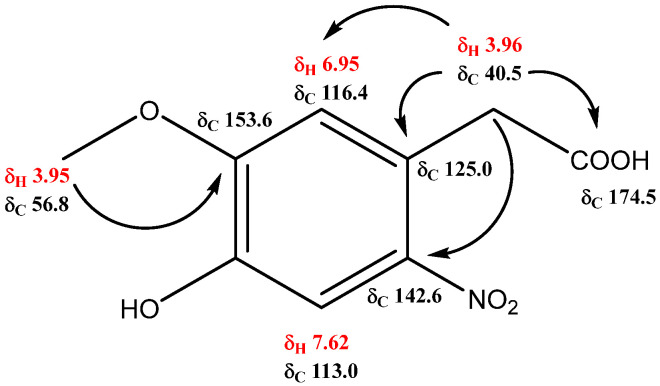
Structure assignment for 6-nitrohomovanillic acid.

The structural determination of 6-nitrohomovanillic acid evidenced in the proton spectrum that the methylene signal (singlet) overlaps with that of the methoxyl group at δ 3.95 (Figure 4). The presence of two distinct signals was confirmed by the HSQC spectrum (Figure 5). Correlation analysis of mono- and bi-dimensional spectra led to the structure assignment for 6-nitrohomovanillic acid (Figure 6).

**Figure 7:**
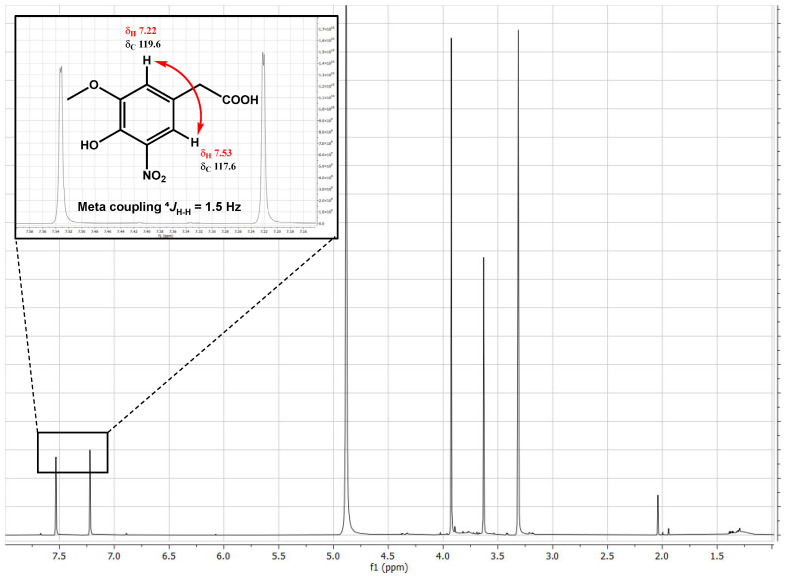
^1^H-NMR of 5-nitrohomovanillic acid in CD_3_OD.

**Figure 8:**
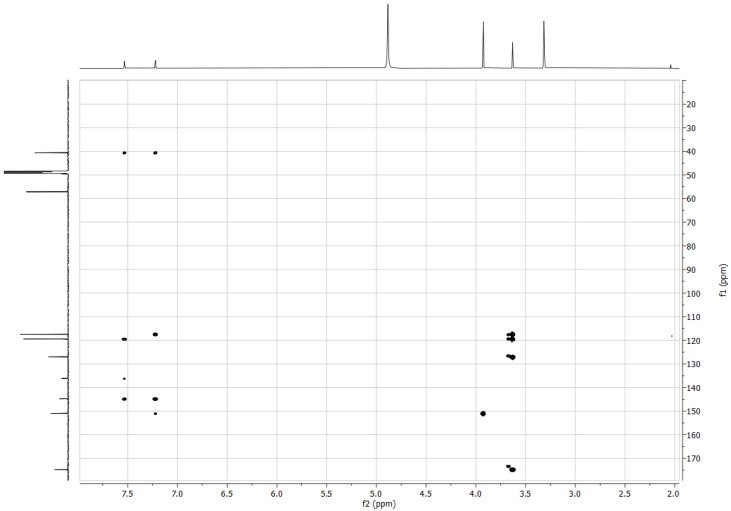
HMBC of 5-nitrohomovanillic acid in CD_3_OD.

**Figure 9:**
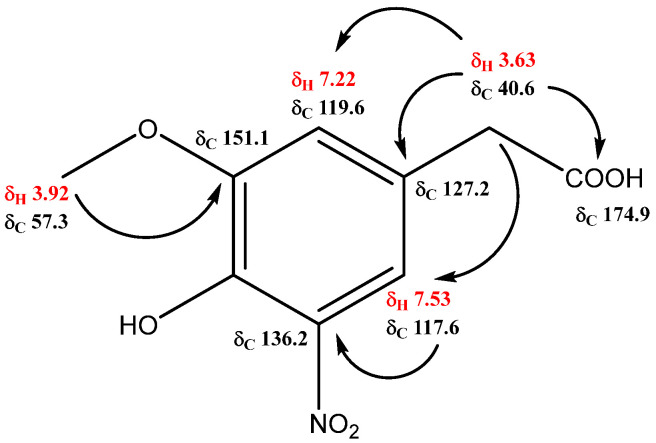
Structure assignment for 5-nitrohomovanillic acid.

When 5-nitrohomovanillic acid was investigated in NMR studies, we noticed the meta coupling of the aromatic protons in the proton spectrum (Figure 7). In this case, it was at δ_H_ 7.22 and 7.53 (^4^*J*_H-H_ = 1.5 Hz). Through a combination with HMBC NMR spectra (Figure 8), we defined the structure assignment (Figure 9).

Full-scan HRMS and HRMS/MS experiments were carried out to investigate the ionization behavior and the fragmentation pattern of the newly synthesized molecules, respectively.

Standard solutions of 6-ND, 6-NHVA, and 5-NHVA at a concentration level of 10 ppm (10 μg/mL) were used to optimize the MS source parameters while operating in positive ionization mode for 6-ND and in negative ionization mode for 6-NHVA and 5-NHVA.

**Figure 10:**
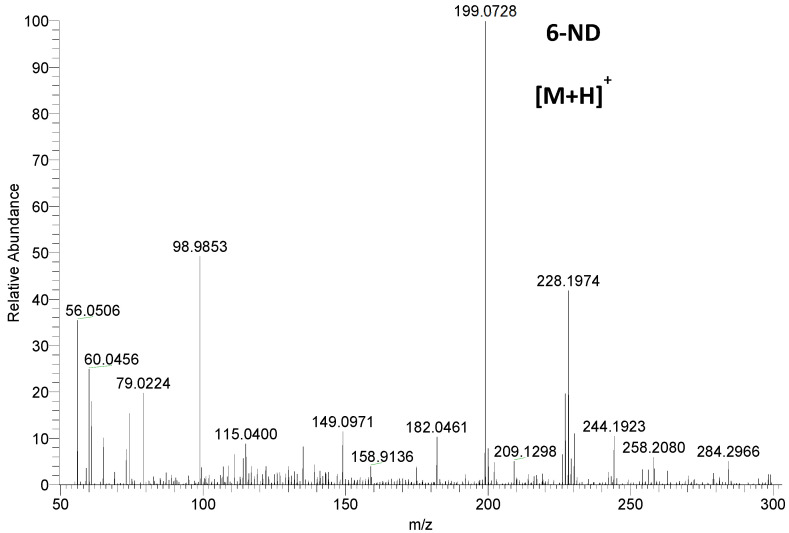
Full-Scan HRMS in the mass range *m*/*z* 50–300 (positive ion mode) of 6-nitrodopamine (6-ND).

The full-scan HRMS spectra of 6-ND (Figure 10) showed the characteristic and dominant [M + H]^+^ ion at *m*/*z* 199.0728 (C_8_H_11_N_2_O_4_, RDBE = 4.5, Δ = 7.167 ppm).

**Figure 11:**
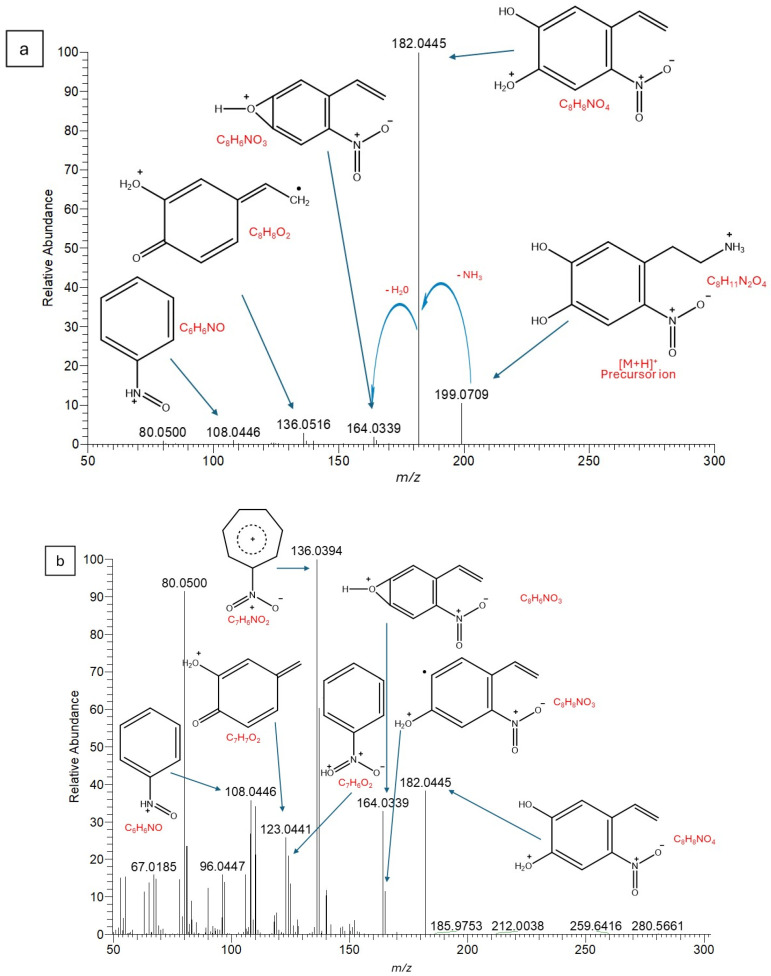
HRMS/MS of the [M + H]^+^ ion of 6-nitrodopamine (6-ND) acquired in the mass range of 50–300 at different collision energies, namely (**a**) CE = 10% and (**b**) CE = 25%. Chemical structures of fragment ions are tentatively proposed.

The HRMS/MS spectra of 6-ND were acquired at the collision energy (CE) values of 10% and 25%, using the [M + H]^+^ ion as precursor. Figure 11 shows the obtained HRMS/MS spectra of 6-ND, along with proposed structures for each observed fragment ion. Elemental composition of each fragment, together with RDBE and mass tolerance (ppm) used in ion assignment, is reported in Table 1.

The HR-MS/MS spectrum of 6-ND at CE 10% (Figure 11a) showed an [M + H-NH_3_]^+^ ion at *m*/*z* 182. 0446 as the base peak of the spectrum, followed by a very weak [M + H-NH_3_-H_2_O]^+^ ion, and a barely visible protonated nitrosobenzene fragment. 6-ND formed a radical cation at *m*/*z* 136.0516 (C_8_H_8_O_2_, RDBE = 5.0) at the 10% CE.

The fragment ion at *m*/*z* 136.0394, tentatively assigned to the nitrotropilium fragment, dominated the HR-MS/MS spectrum of 6-ND at 25% CE (Figure 11b).

**Figure 12:**
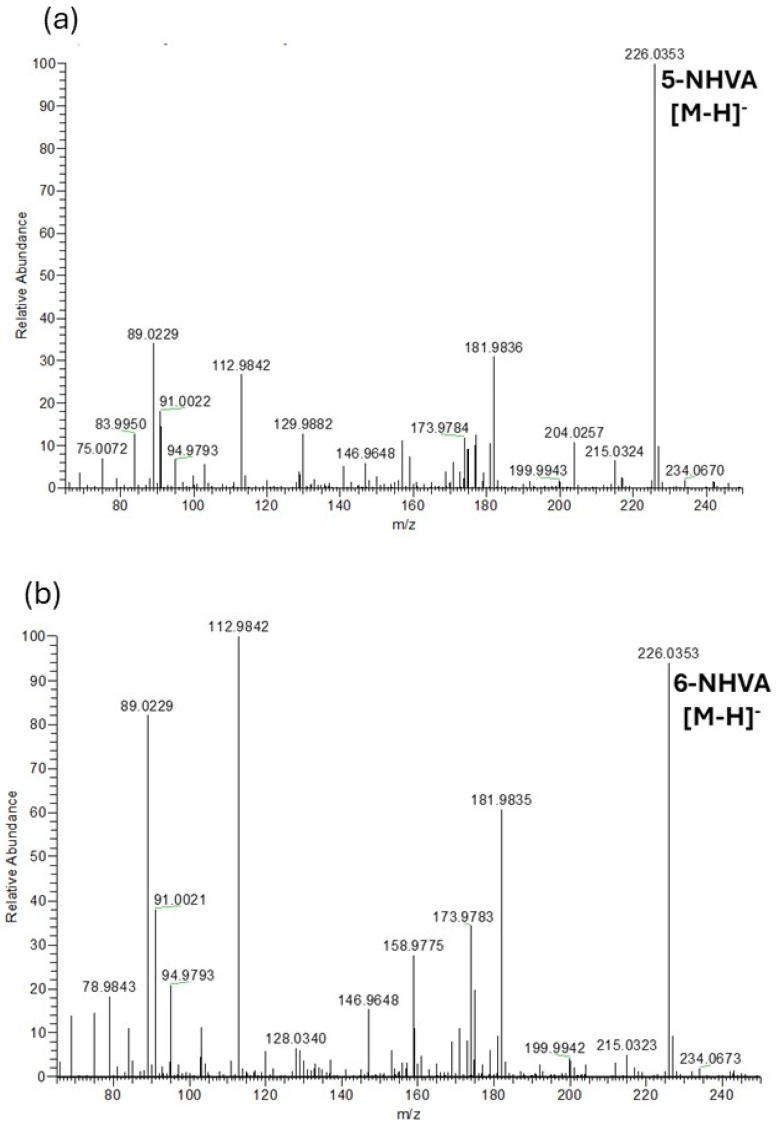
Full-scan HRMS of (**a**) 5-nitrohomovanillic acid (5-NHVA) and (**b**) 6-nitrohomovanillic acid (6-NHVA) acquired in the mass range 50–250 in negative ion mode.

The full-scan HRMS spectra of the 5- and 6-NHVA (Figure 12) showed the characteristic and dominant [M-H]^−^ ions at *m*/*z* 226.0353 (C_9_H_8_NO_6_, RDBE = 6.5, Δ = 3.082 ppm).

**Figure 13:**
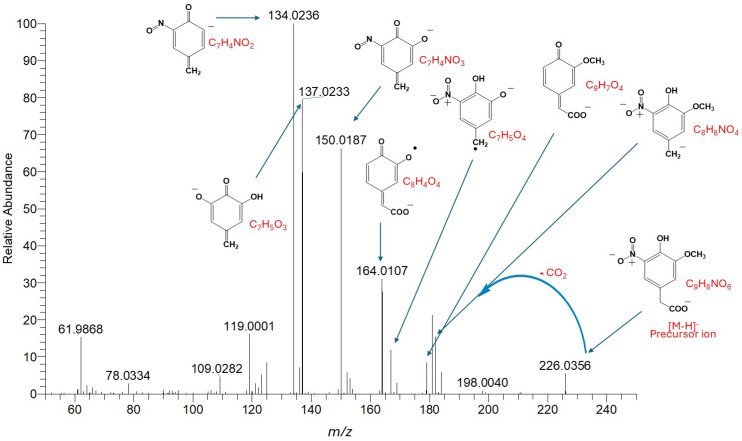
HRMS/MS of the [M-H]^−^ ion of 5-nitrohomovanillic acid (5-NHVA) acquired in the mass range 50–250 at CE = 16%. Chemical structures of fragment ions are tentatively proposed.

**Figure 14:**
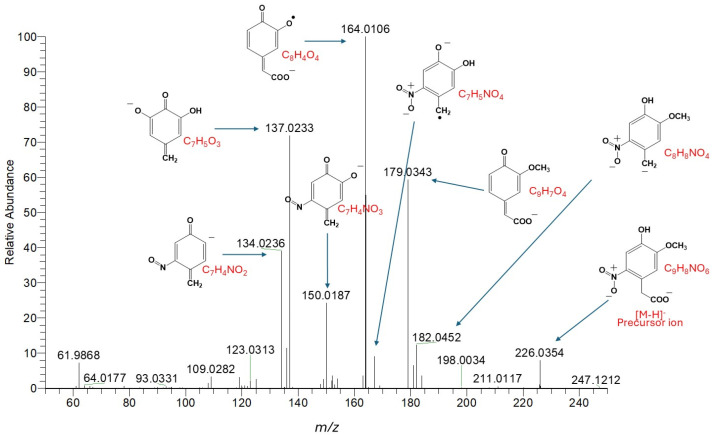
HRMS/MS of the [M-H]^−^ ion of 6-nitrohomovanillic acid (6-NHVA) acquired in the mass range *m*/*z* 50–250 at CE = 14%. Chemical structures of fragment ions are tentatively proposed.

The HRMS/MS spectra of 5-NHVA and 6-NHVA were acquired at CE values of 16% and 14%, respectively, using the [M-H]^−^ ion of each compound as a precursor. Figure 13 and Figure 14 report HRMS/MS spectra of [M-H]^−^ ions of 5- and 6-NHVA, respectively, along with proposed structures for the observed fragment ions. The elemental composition of each fragment is reported in Table 2 and Table 3, respectively, together with RDBE and error (ppm).

Analyzing the fragment ions contained in the HR-MS/MS spectrum of [M-H]^−^ ion of 5-NHVA (Figure 13) in decreasing order of *m*/*z*, a fragment at *m*/*z* 182.0453 (C_8_H_8_NO_4_, RDBE = 5.5), due to the neutral loss of CO_2_ from the precursor, was observed. Loss of HNO_2_ from the precursor [M-H]^−^ ion of 5-NHVA originated the fragment at *m*/*z* 179.0343 (C_9_H_7_O_4_ RDBE = 6.5). Two radical anions at *m*/*z* 167.0216 and 164.0107 likely derived from the above-mentioned fragments [M-H-CO_2_]^−^ and [M-H-HNO_2_]^−^, respectively, by the loss of a methyl group. Intense fragments were observed at *m*/*z* 150.0187 and at 134.0236 (base peak) and tentatively assigned to nitrosoderivatives of 5-NHVA. Finally, the fragment at *m*/*z* 137.0233 might be due to a loss of NO from *m*/*z* 167.0216.

Comparison between HR-MS/MS spectra of 5-NHVA (CE = 16%) and 6-NHVA (CE = 14%) showed that fragmentation patterns of the regioisomers were actually superimposable in terms of absolute *m*/*z* values, differing only for the relative intensity of the observed fragments. By way of example, 6-NHVA fragmented, forming the ion at *m*/*z* 164.0106 as the base peak (Figure 14), whereas 5-NHVA formed the ion at *m*/*z* 134.0236 as the base peak. This MS behavior, common to regioisomers and even to diasteroisomers of many organic compounds, might be exploited to selectively detect 5-NHVA and 6-NHVA in biological matrices, even using mass spectrometers operating at unit resolution, such as triple-quadrupole MS or ion traps. Complete ion assignment for 6-NHVA is reported in Table 3.

### 2.2. 6-NHVA, 5-NHVA, and HVA Levels in Human Amniotic Fluid

In a sample of human amniotic fluid, 6-NHVA (0.64 ng/mL), 5-NHVA (0.47 ng/mL), and HVA (1.91 μg/mL) were detected by LC-MS/MS. The chromatograms are reported in the Supplementary Materials (Figure S11). This is the first time that these two novel metabolites have been identified in a biological system. These data suggest the hypothesis that the pathway that generates 5-NHVA and 6-NHVA from 5-ND and 6-ND, respectively, may involve the same enzymatic systems known for dopamine metabolism [1,2]. This hypothesis is graphically summarized in Scheme S1.

## 3. Materials and Methods

### 3.1. Chemistry

Homovanillic acid (HVA), deuterated homovanillic acid (HVA-d3), and 6-nitrodopamine (6-ND) were acquired from Toronto Research Chemicals (TRC, Toronto, ON, Canada). Formic acid and acetic acid were obtained from Sigma-Aldrich Chemicals Co. (St. Louis, MO, USA). Acetonitrile (MeCN) and methanol (MeOH) were supplied by J.T. Baker (Phillipsburg, NJ, USA). Ultrapure water was prepared in-house using the Synergy UV^®^ purification system (Merck Millipore, Molsheim, France). All the other commercial reagents and solvents were purchased from Merck (Darmstadt, Germany). Reactions were stirred at 400 rpm by a Heidolph MR Hei-Standard magnetic stirrer (Heidolph Scientific Products GmbH, Schwabach, Germany). Solutions were concentrated under vacuum using a Buchi R-114 rotary evaporator (BÜCHI Labortechnik AG, Flawil, Switzerland). All reactions were monitored by using TLC silica gel 60 F254 plates (Merck, Darmstadt, Germany) with a fluorescent indicator, visualizing the spots under UV light (Delchimica Scientific Glassware, Naples, Italy) at 254 nm wavelength. The isolation of the synthesized compounds was carried out by preparative HPLC. The purity of the isolated compounds **I** and **II** was determined by analytical RP-HPLC, which used a Phenomenex Biozen 3.6 μM Intact XB-C8 50 × 4.6 mm column (Phenomenex, Torrance, CA, USA). The column was connected to a Rheodyne model 7725 injector (Thermo Fisher Scientific, Waltham, MA, USA) a Shimadzu-10ADsp HPLC system (Shimadzu, Kyoto, Japan), and a Shimadzu SPD-20A/SPD-20AV UV−vis detector (Shimadzu, Kyoto, Japan) set to 254 nm. The analytical determination employed solvent A, water with 0.1% TFA, and solvent B, acetonitrile with 0.1% TFA. The following gradient was used: t 0 min = 0% B, t 7 min = 0% B, t 10 min = 40% B, t 12 min = 100% B, t 14 min = 0% B (Total Run Time 14.00 min), with 2 min of re-equilibration time. The sample was dissolved as follows: 1 mg in 1 mL of H_2_O, successively diluted to 50 µg/mL. An injection of 10 µL was repeated in triplicate. Purity was ≥99% and was further confirmed by NMR. Analytical chromatograms of 6-ND, 6-NHVA, and 5-NHVA are reported in Figures S1–S4. Reversed-phase SPE cartridges (Strata-X 33 μm Polymeric Reversed Phase) were bought from Phenomenex (Torrance, CA, USA). Melting points were measured on chromatographically purified material using a Buchi Melting Point B-540 instrument (BÜCHI Labortechnik AG, Flawil, Switzerland) and reported as uncorrected values. All NMR spectra were obtained using a Bruker Avance NEO 700 MHz instrument (Bruker Scientific, Manning Park Billerica, MA, USA). The chemical shifts were reported in ppm and were referenced to the residual solvent signal. For an accurate measurement of the coupling constants, the 1D ^1^H NMR spectra were transformed at 64 K points (digital resolution: 0.09 Hz). Two- and three-bond ^1^H–^13^C connectivities were determined by gradient 2D HMBC experiments optimized for a ^2,3^*J* of 8 Hz. ^3^*J*_H–H_ values and ^4^*J*_H–H_ values were extracted from 1D ^1^H NMR. ^13^C-NMR and HMQC for 6-ND (Figures S5 and S6, respectively), ^13^C-NMR and HSQC for 5-NHVA (Figures S7 and S8, respectively), and ^13^C-NMR and HMBC for 6-NHVA (Figures S9 and S10, respectively) are reported in the Supplementary Materials.

High-resolution MS (HRMS) and MS/MS experiments were carried out on a Q-Exactive Orbitrap MS system (ID 4C52620A8D67) equipped with an electrospray ionization (ESI) Ion-Max^TM^ source (Thermo Fisher, San Josè, CA, USA). Solutions of 6-ND, 6-NHVA, and 5-NHVA were prepared by dissolving 1 mg of each compound in 1 mL of ACN-H_2_O 1:1 (*v*/*v*) with 0.1% acetic acid, further diluted to 10 μg/mL (10 ppm) with the same solvent mixture, and directly infused in HRMS through a syringe pump operating at 10 µL/min.

Full-scan HRMS of 6-ND was acquired in positive ion mode in the mass range *m*/*z* 50–300 using the following source parameters: capillary temperature = 320 °C, sheath gas flow = 10 (arbitrary units), auxiliary gas = 0 (arbitrary units), spray voltage = +4200 V. Full-scan HRMS of 6-HVA and 5-NHVA were acquired in negative ion mode in the mass range *m*/*z* 50–250 using the following source parameters: capillary temperature = 320 °C, sheath gas flow = 19 (arbitrary units), auxiliary gas = 2 (arbitrary units), spray voltage = −3000 V. HRMS/MS experiments were recorded using high-energy collision-induced dissociation (HCD) in the mass range *m*/*z* 50–300 for 6-ND and *m*/*z* 65–250 for 6-NHVA and 5-NHVA using collision energies in the range of 10–25%. The [M + H]^+^ ion at *m*/*z* 199.0 was selected as the precursor for 6-ND (resolving power FWHM at *m*/*z* 199 = 40,000), whereas the [M - H]^−^ ion at *m*/*z* 226.0 was selected as the precursor for 6-NHVA and 5-NHVA (resolving power FWHM at *m*/*z* 226 = 36,300). Data processing was carried out using Xcalibur software 2.9–290033. The following constraints—N 0 to 10; O 0 to 15, C 0 to 30, H 0 to 60—and a mass tolerance of 5 ppm were used in ion assignment. Ring Double Bond Equivalent values were calculated according to the equation by Badertscher et al. [24], and compared with those automatically calculated by the software. Notably, this formula can be used with charged species: RDB gives half-integer results for even-electron ions and integer results for odd-electron ions.

$$DU=1+\frac{1}{2}\;\sum n\;(v-2)$$

For LC-MS/MS in the development of the analytical method for the determination of 6-NHVA and 5-NHVA in human amniotic fluid, we used a Nexera HPLC system consisting of an autoinjector model SIL-30AC, an LC-30AD binary pump, and a CTO-20AC column oven coupled to an LCMS-8060 triple-quadrupole mass spectrometer (MS/MS) (Shimadzu, Kyoto, Japan).

#### 3.1.1. Synthesis of 2-(4-Hydroxy-5-methoxy-2-nitrophenyl)acetic Acid (6-Nitrohomovanillic Acid, **I**, 6-NHVA)

Nitronium tetrafluoroborate (0.80 g, 6.04 mmol) was added to a solution of 4-hydroxy-3-methoxyphenylacetic acid (1.00 g, 5.49 mmol) in dry ACN (10 mL). The mixture was stirred at room temperature for 2 h under a N_2_ atmosphere. The solvent was removed under vacuum, and the crude product was purified by preparative RP-HPLC using H_2_O + 0.1% TFA (A) and ACN + 0.1% TFA (B) as mobile phases. The purification was performed through a linear gradient of 5–70% B in 20 min. The purity of the compound was assessed by analytical RP-HPLC, employing the same solvents and method. Analytical HPLC: *t*R 13.89 min. Compound **I**, brown solid (0.85 g, 68%). ^1^H NMR (700 MHz, CD_3_OD): δ 7.62 (s, 1H), 6.95 (s, 1H), 3.96 (s, 2H), 3.95 (s, 3H). ^13^C NMR (175 MHz, CD_3_OD): δ 40.5 (-CH_2_-), 56.8 (-OCH_3_), 113.0, 116.4, 125.0, 142.6 (-CNO_2_), 147.2, 153.6, 174.5 (-COOH). ESI-MS *m*/*z* [M-H]^−^ calculated for C_9_H_9_NO_6_ 226.0357, found = 226.0354. m.p. 155–156.5 °C (dec.).

#### 3.1.2. Synthesis of 2-(4-Hydroxy-3-methoxy-5-nitrophenyl)acetic Acid (5-Nitrohomovanillic Acid, **II**, 5-NHVA)

4-Hydroxy-3-methoxyphenylacetic acid (1.00 g, 5.49 mmol) was solubilized in ethyl acetate (10 mL), and HNO_3_ 65% (0.38 mL, 5.49 mmol) and zinc chloride (0.75 g, 5.49 mmol) were added. The mixture was stirred at room temperature, and after three hours, another equivalent of HNO_3_ 65% was added. The reaction was left to stir overnight. The solvent was evaporated, and the crude material was purified by preparative RP-HPLC using H_2_O + 0.1% TFA (A) and ACN + 0.1% TFA (B) as mobile phases. The purification was performed through a linear gradient of 5–70% B in 20 min. Purity of the compound was assessed by analytical RP-HPLC, employing the same solvents and method. Analytical HPLC: *t*R 15.39 min. Compound **II**, yellow solid (1.10 g, 89%). ^1^H NMR (700 MHz, CD_3_OD): δ 7.53 (d, ^4^*J*_H–H_ = 1.5 Hz, 1H), 7.22 (d, ^4^*J*_H–H_ = 1.5 Hz, 1H), 3.92 (s, 3H), 3.63 (s, 2H). ^13^C NMR (175 MHz, CD_3_OD): δ 40.6 (-CH_2_-), 57.3 (-OCH_3_), 117.6, 119.6, 127.2, 136.2 (-CNO_2_), 144.9, 151.1, 174.9 (-COOH). ESI-MS *m*/*z* [M-H]^−^ calculated for C_9_H_9_NO_6_ 226.0357, found = 226.0356. m.p. 220–222 °C (dec.).

### 3.2. Analytical Procedure for the Determination of 6-NHVA, 5-NHVA, and HVA in Human Amniotic Fluid

An amniotic fluid sample was acquired directly during a cesarean section by aspirating the fluid immediately after the uterus was opened, but before the fetus was extracted. The procedure was approved by the local Research Ethics Committee, Institute of Biomedical Sciences, University of São Paulo (ICB-USP; Project: GDN 002/24, CAAE: 80198424.2.000.5467), and the subject signed an informed consent form. The analytes were extracted via solid phase extraction, and the residues were analyzed using a Nexera HPLC system consisting of an autoinjector model SIL-30AC, an LC-30AD binary pump, and a CTO-20AC column oven coupled to an LCMS-8060 triple-quadrupole mass spectrometer (MS/MS) (Shimadzu, Kyoto, Japan). Separation was achieved on a 150 × 3 mm Shim-pack GIST C18-AQ column at 55 °C, using an isocratic mobile phase of 65% mobile phase A (0.05% acetic acid in water) and 35% mobile phase B (90% methanol/10% water) at a flow rate of 0.5 mL/min. The system operated in negative electrospray ionization (ESI) and multiple reaction monitoring (MRM) mode: HVA (*m*/*z*: 181.1 >> 137.3; retention time: 4.3 min); HVA-d3 (*m*/*z*: 184.1 >> 140.1; retention time: 4.25 min); 6-NHVA (*m*/*z*: 225.95 >> 179.25; retention time: 4.9 min); and 5-NHVA (*m*/*z*: 225.95 >> 134.25; retention time: 7.2 min). The full validation will be reported in due course.

## 4. Conclusions

The aim of the present study was to synthesize 6-NHVA and 5-NHVA as useful probes for the investigation of their levels in biological fluids. Two important results were achieved. The first was the optimization of the synthetic procedures for 6-NHVA and 5-NHVA, which to date represent the best experimental conditions. Indeed, the products were obtained in a single step, and our procedures allow higher yields than any procedure published so far for these molecules. The second was the identification of these two novel metabolites in human amniotic fluid.

NMR studies confirmed position 6 of the nitro group on the aromatic ring of 6-ND and 6-NHVA and position 5 for 5-NHVA.

**Figure 15:**
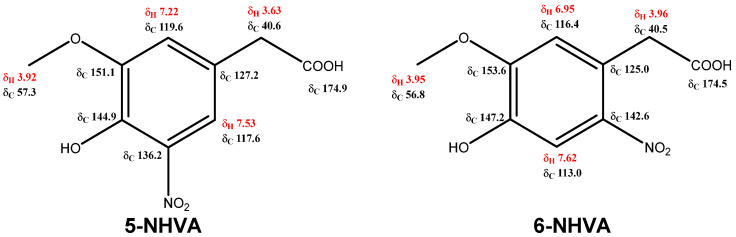
Chemical shift comparison between 5-NHVA and 6-NHVA.

Figure 15 shows the chemical structures of 5-NHVA and 6-NHVA, highlighting differences in their NMR spectra (δ_H_ and δ_C_). The chemical shift values, shown in red for protons and black for carbons, demonstrate how the position of the NO_2_ group influences the electronic distribution of the aromatic ring. The differences in chemical shifts confirm that introducing NO_2_ at position 6 significantly alters the chemical environment compared to position 5.

High-resolution mass spectrometry experiments allowed us to assign molecular formulae and unsaturation degrees to 6-ND (C_8_H_10_N_2_O_4_, RDBE = 5), which ionized in positive ion mode, as well as to 5- and 6-NHVA (C_9_H_9_NO_6_, RDBE = 6), which ionized in negative ion mode. The accuracy of mass measurements was below 10 ppm in all cases.

The HRMS/MS spectra of 5-NHVA and 6-NHVA contained several diagnostic fragment ions that allowed us to confirm their chemical structures. Discrimination between the regioisomers was possible by acquiring HRMS/MS spectra, as the fragmentation patterns of the molecules were the same in terms of exact masses, differing only in relative ratios of the observed fragment ions. In particular, in the HRMS/MS spectra of 5- and 6-NHVA acquired in negative ion mode, we noticed the following fragmentation pattern that presented the order of intensity for the four most diagnostic ions as follows: *m*/*z* 134.0236 > 137.0233 > 150.0187 > 164.0107 for 5-NHVA and *m*/*z* 164.0106 > 137.0233 > 179.0343 > 134.0236 for 6-NHVA.

In this paper, we report the identification of 6-NHVA and 5-NHVA for the first time in a biological system. Although the concentrations were several thousand times lower when compared to HVA, this finding does not indicate that 6-ND and 5-ND should undergo a different metabolic pathway than dopamine. Indeed, 6-ND is ten thousand times more potent as a chronotropic agent than dopamine in the rat isolated atria.

## Figures and Tables

**Table 1:** Ion assignment of the fragments contained in the HRMS/MS spectra (CE 10% and CE 25%) of 6-nitrodopamine. Elemental formulae of the monoisotopic ion peaks (*m*/*z*) are reported with Ring Double Bond Equivalents (RDBEs) and errors (ppm). Base peaks are reported in bold.

Ion, *m*/*z*	Formula	RDBE	Δ, ppm
199.0709 ^a^	C_8_H_11_N_2_O_4_	4.5	−2.076
**182.0446 ^a,b^**	**C_8_H_8_NO_4_**	**5.5**	**−1.232**
165.0418 ^b^	C_8_H_7_NO_3_	6.0	−1.482
164.0339 ^a,b^	C_8_H_6_NO_3_	6.5	−2.192
137.0469 ^b^	C_7_H_7_NO_2_	5.0	−1.605
136.0516 ^a^	C_8_H_8_O_2_	5.0	−1.992
**136.0394 ^b^**	**C_7_H_6_NO_2_**	**5.5**	**0.698**
124.0393 ^b^	C_6_H_6_NO_2_	4.5	0.121
123.0441 ^b^	C_7_H_7_O_2_	4.5	0.114
108.0446 ^a,b^	C_6_H_6_NO	4.5	1.662

^a^ fragment ions observed at CE = 10%; ^b^ fragment ions observed at CE = 25%.

**Table 2:** Ion assignment of the fragments contained in the HRMS/MS spectrum (CE 16%) of 5-nitrohomovanillic acid. Elemental formulae of the monoisotopic ion peaks (*m*/*z*) are reported with Ring Double Bond Equivalents (RDBEs) and errors (Δ, ppm). Base peak is reported in bold.

Ion, *m*/*z*	Formula	RDBE	Δ, ppm
226.0356	C_9_H_8_NO_6_	6.5	4.365
182.0453	C_8_H_8_NO_4_	5.5	2.613
179.0343	C_9_H_7_O_4_	6.5	2.596
167.0216	C_7_H_5_NO_4_	6.0	1.682
164.0107	C_8_H_4_O_4_	7.0	1.463
150.0187	C_7_H_4_NO_3_	6.5	0.736
137.0233	C_7_H_5_O_3_	5.5	0.069
**134.0236**	**C_7_H_4_NO_2_**	**6.5**	**−0.111**

**Table 3:** Ion assignment of the fragments contained in the HRMS/MS spectrum (CE 14%) of 6-nitrohomovanillic acid. Elemental formulae of the monoisotopic ion peaks (*m*/*z*) are reported with Ring Double Bond Equivalents (RDBEs) and errors (Δ, ppm). Base peak is reported in bold.

Ion, *m*/*z*	Formula	RDBE	Δ, ppm
226.0354	C_9_H_8_NO_6_	6.5	3.613
182.0452	C_8_H_8_NO_4_	5.5	2.449
179.0343	C_9_H_7_O_4_	6.5	2.149
167.0216	C_7_H_5_NO_4_	6.0	1.203
**164.0106**	**C_8_H_4_O_4_**	**7.0**	**1.158**
150.0187	C_7_H_4_NO_3_	6.5	0.603
137.0233	C_7_H_5_O_3_	5.5	−0.150
134.0236	C_7_H_4_NO_2_	6.5	−0.261

## Data Availability

Data are available on request from the corresponding author.

## Supplementary Materials

The following supporting information can be downloaded at: https://www.mdpi.com/article/10.3390/molecules30204096/s1, Figure S1: HPLC chromatograms of 6-ND, Figure S2: HPLC chromatograms of 5-NHVA, Figure S3: HPLC chromatograms of 6-NHVA, Figure S4: HPLC chromatogram of 5-NHVA and 6-NHVA in mixture. Figure S5: ^13^C NMR spectra in CD_3_OD of 6-ND, Figure S6: HMBC spectra in CD_3_OD of 6-ND, Figure S7: ^13^C NMR spectra in CD_3_OD of 5-NHVA, Figure S8: HSQC in CD3OD of 5-NHVA, Figure S9: ^13^C NMR spectra in CD_3_OD of 6-NHVA, Figure S10: HMBC in CD_3_OD of 6-NHVA, Figure S11: HVA, 6-NHVA and 5-NHVA identification by LC-MS/MS in human amniotic fluid sample, Scheme S1: Dopamine metabolism and proposed 6-ND and 5-ND metabolic pathways.
