# Supplementary material for: Improved Synthesis of 5-Nitrohomovanillic Acid and 6-Nitrohomovanillic Acid as Probes for Metabolism Studies of Endothelium-Derived Dopamines: Identification in Human Amniotic Fluid
Source: Molecules. 2025 Oct 15;30(20):4096. doi: 10.3390/molecules30204096 (PMC12566566; doi:10.3390/molecules30204096)
Supplement: Supplementary file 1 [file molecules-30-04096-s001.zip › molecules-3881117-supplementary.pdf]

Supporting Information

Improved Synthesis of 5-Nitrohomovanillic Acid and 6-Nitrohomovanillic Acid as Probes for Metabolism Studies of Endothelium-Derived Dopamines: Identification in Human Amniotic Fluid

**Rosa Sparaco [1], Pierfrancesco Cinque [1], Antonia Scognamiglio [1], Stefania Vertuccio [1], Giuseppe Caliendo [1,*], Ferdinando Fiorino [1], Angela Corvino [1], Elisa Magli [2], Elisa Perissutti [1], Vincenzo Santagada [1], Beatrice Severino [1], Giorgia Andreozzi [1], Paolo Luciano [1], Carmela Dell'Aversano [1], Alex Henrique Miller [3], Gilberto De Nucci [3,4]
and Francesco Frecentese [1]**

[1] Department of Pharmacy, University of Naples Federico II, Via D. Montesano 49, 80131 Naples, Italy;
rosa.sparaco@unina.it (R.S.); pierfrancesco.cinque@unina.it (P.C.);
antonia.scognamiglio@unina.it (A.S.); stefania.vertuccio@unina.it (S.V.); fefiorin@unina.it (F.F.);
angela.corvino@unina.it (A.C.);
perissut@unina.it (E.P.); santagad@unina.it (V.S.); bseverin@unina.it (B.S.);
giorgia.andreozzi@unina.it (G.A.); pluciano@unina.it (P.L.); dellaver@unina.it (C.D.);
frecente@unina.it (F.F.)
[2] Department of Public Health, University of Naples Federico II, Via Pansini 5, 80131 Naples, Italy; elisa.magli@unina.it
[3] Department of Pharmacology, Faculty of Medical Sciences, State University of Campinas (UNICAMP), Campinas 13083-888, SP, Brazil; amiller@unicamp.br (A.H.M.); denucci@unicamp.br (G.D.N.)
[4] Department of Pharmacology, Faculdade São Leopoldo Mandic, Campinas 13045-755, SP, Brazil
* Correspondence: caliendo@unina.it; Tel.: +39-081 678649

Content:

Chromatograms of 6-ND, 6-NHVA and 5-NHVA…….…………………..……………pages S2-S5

^{13}C-NMR and HMBC for 6-ND….-………………………………………..…....pages S6-S7

^{13}C-NMR and HSQC for 5-NHVA……………………………………..……..…..pages S8-S9

^{13}C-NMR and HMBC for 6-NHVA…………………………………….…..……..pages S10-S11

HVA, 6-NHVA and 5-NHVA identification by LC-MS/MS in human amniotic fluid sample ………………………………………………………………………………...……..page S12

Dopamine metabolism and proposed 6-ND and 5-ND metabolic pathways………………..page S13

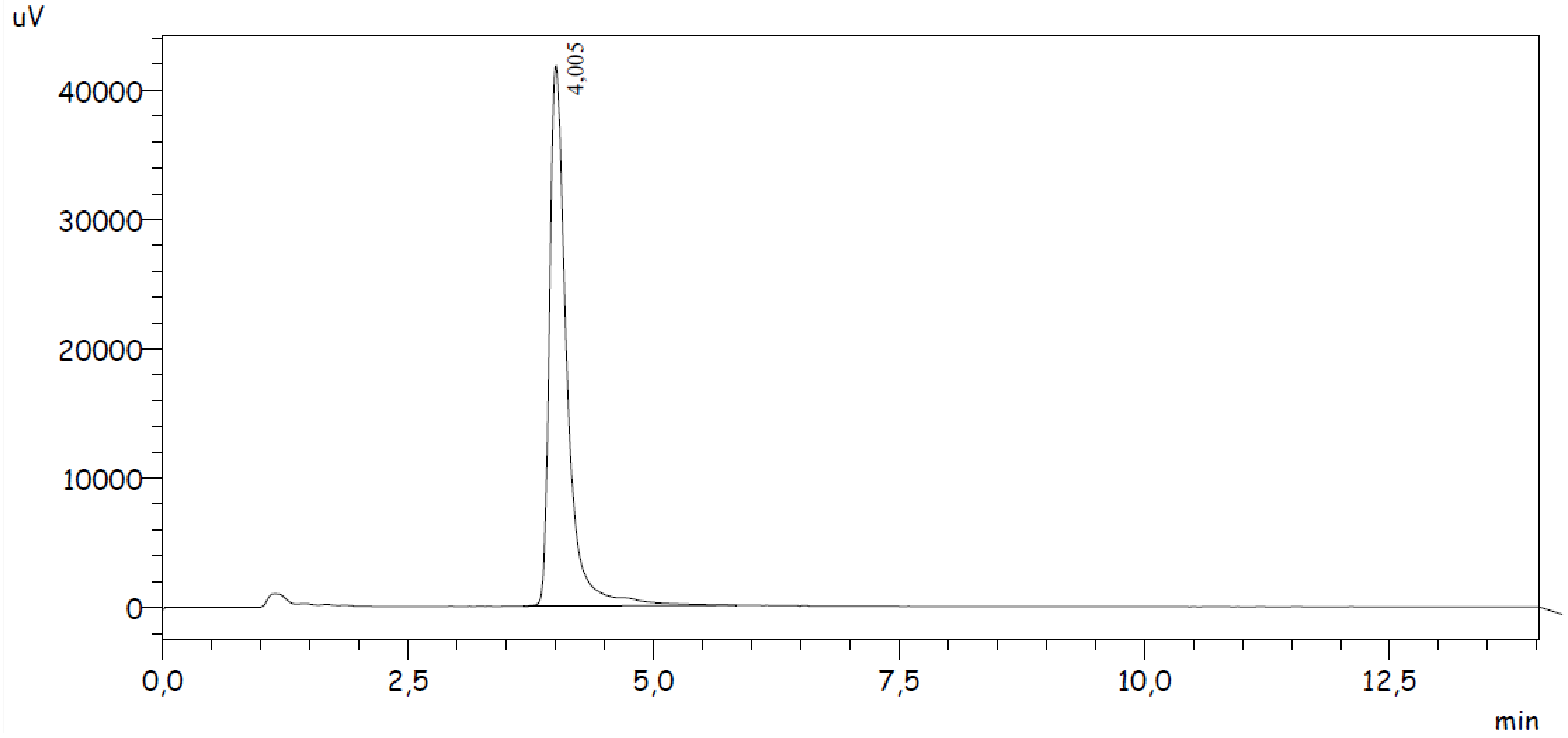

Figure S1. HPLC chromatogram of 6-ND.

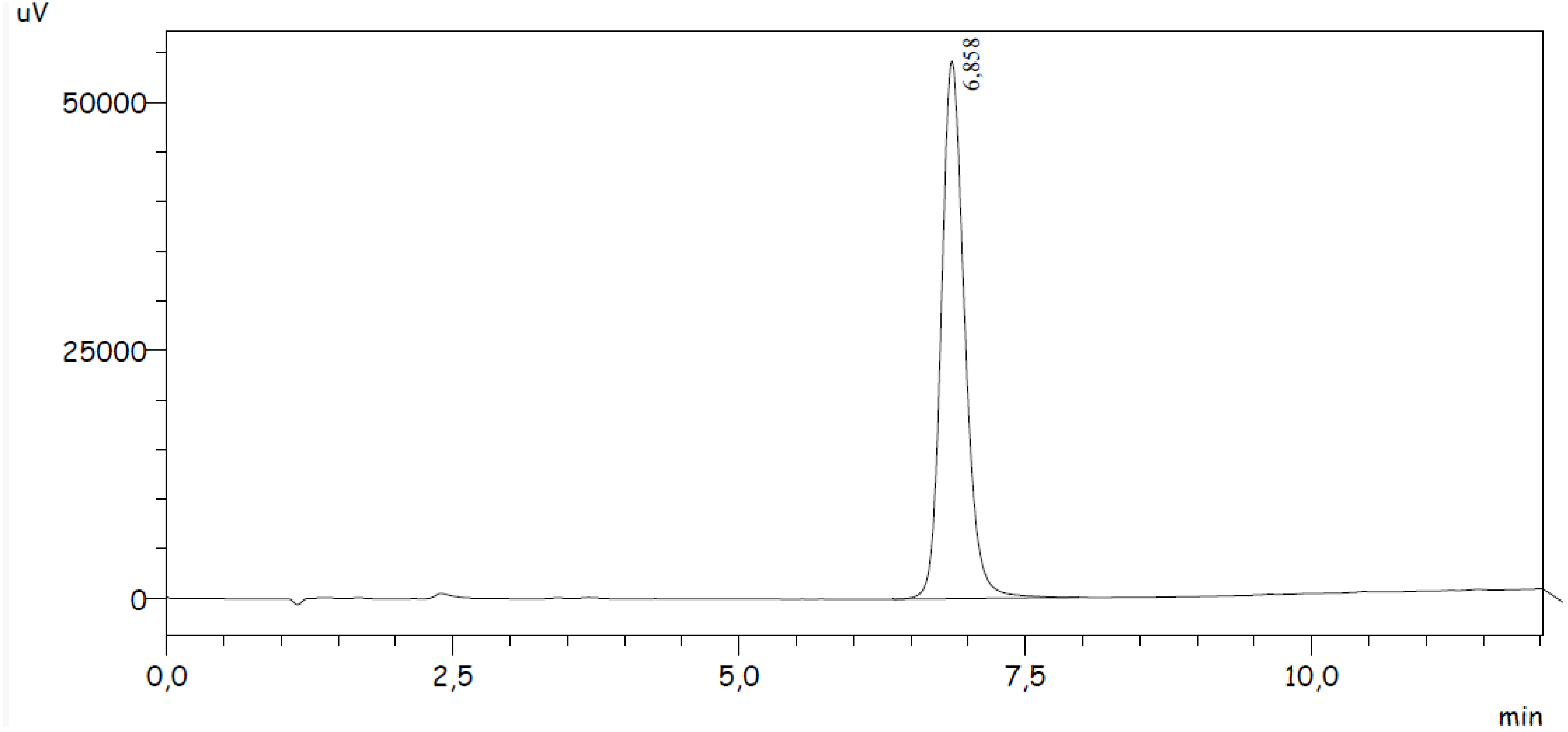

Figure S2. HPLC chromatogram of 5-NHVA.

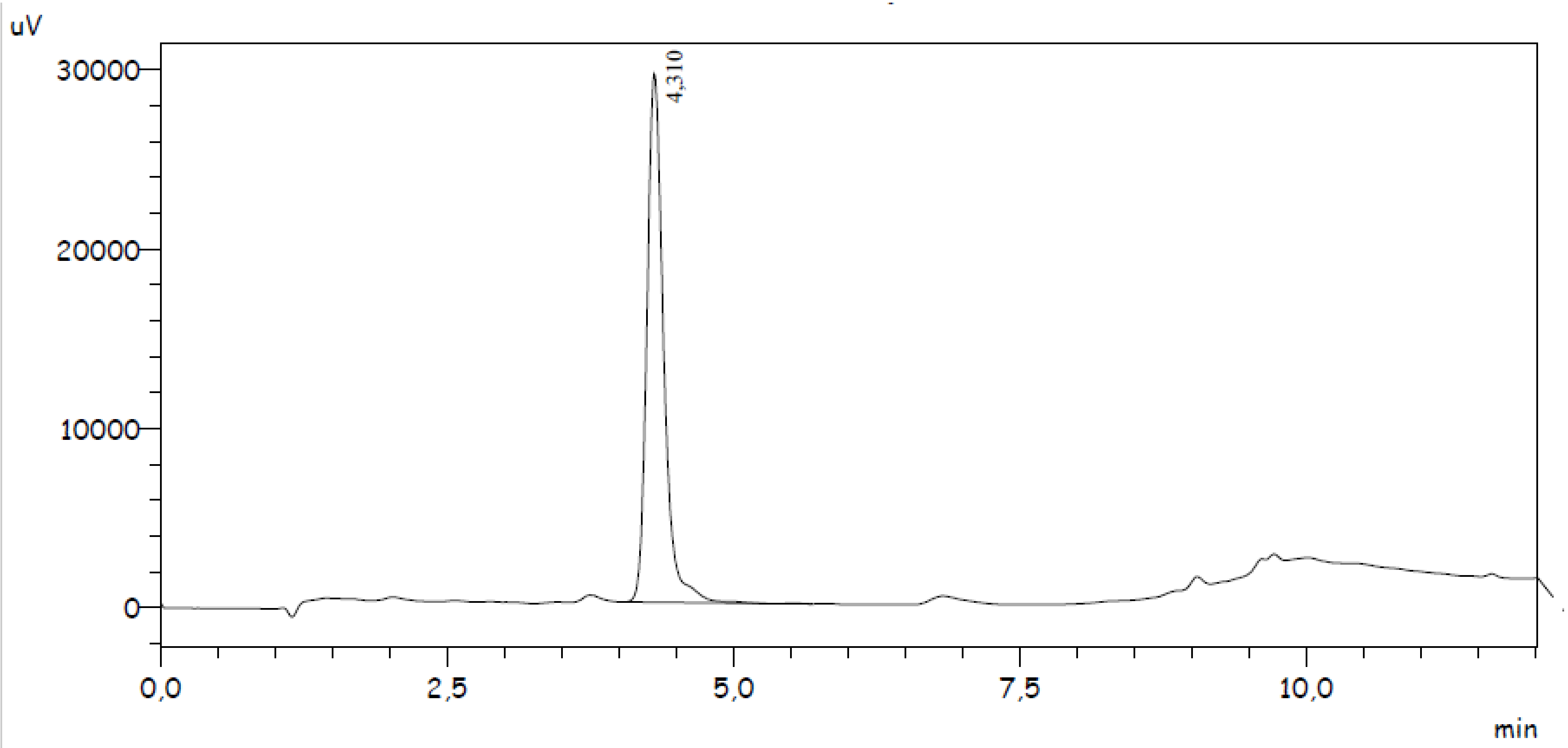

Figure S3. HPLC chromatogram of 6-NHVA.

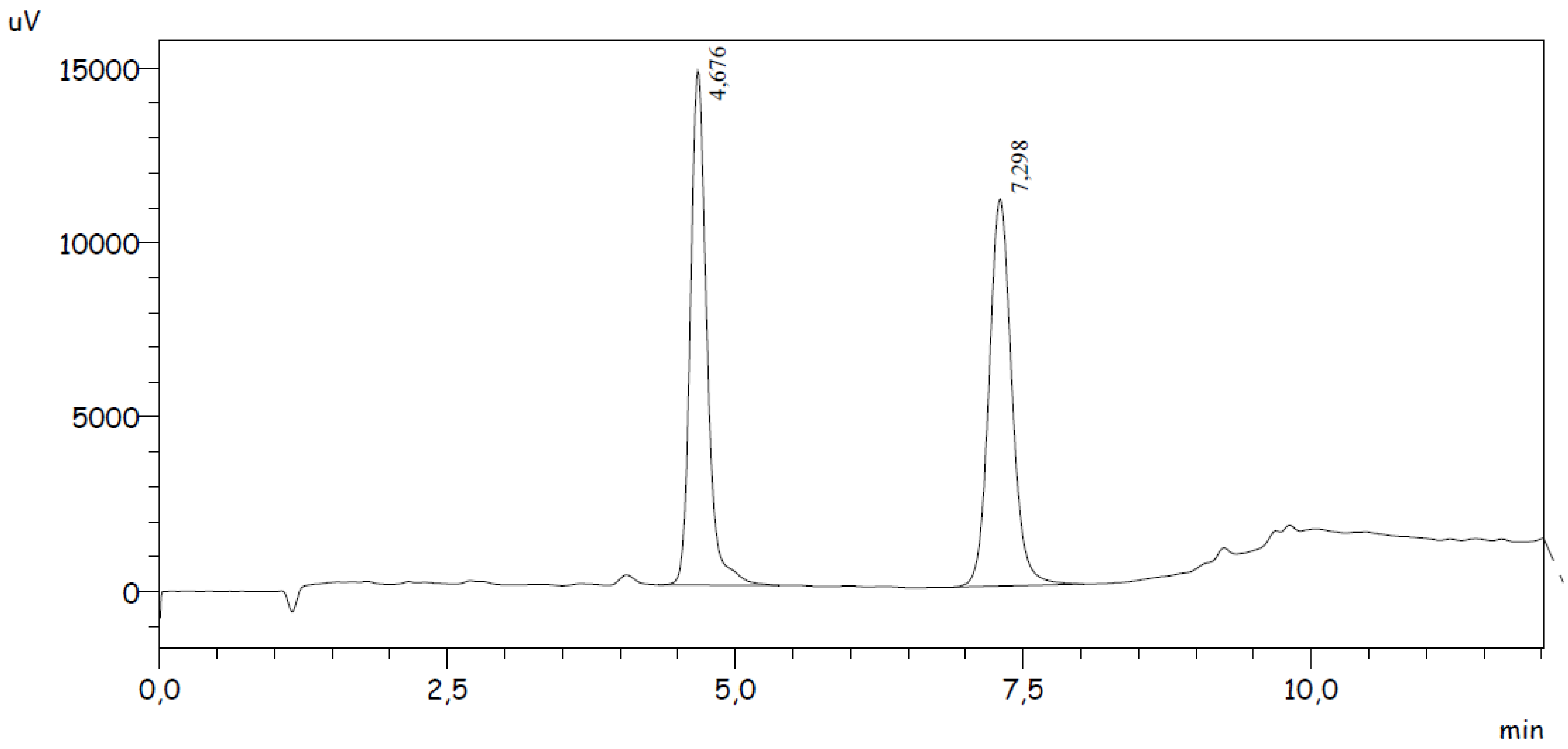

Figure S4. HPLC chromatogram of 5-NHVA and 6-NHVA in mixture.

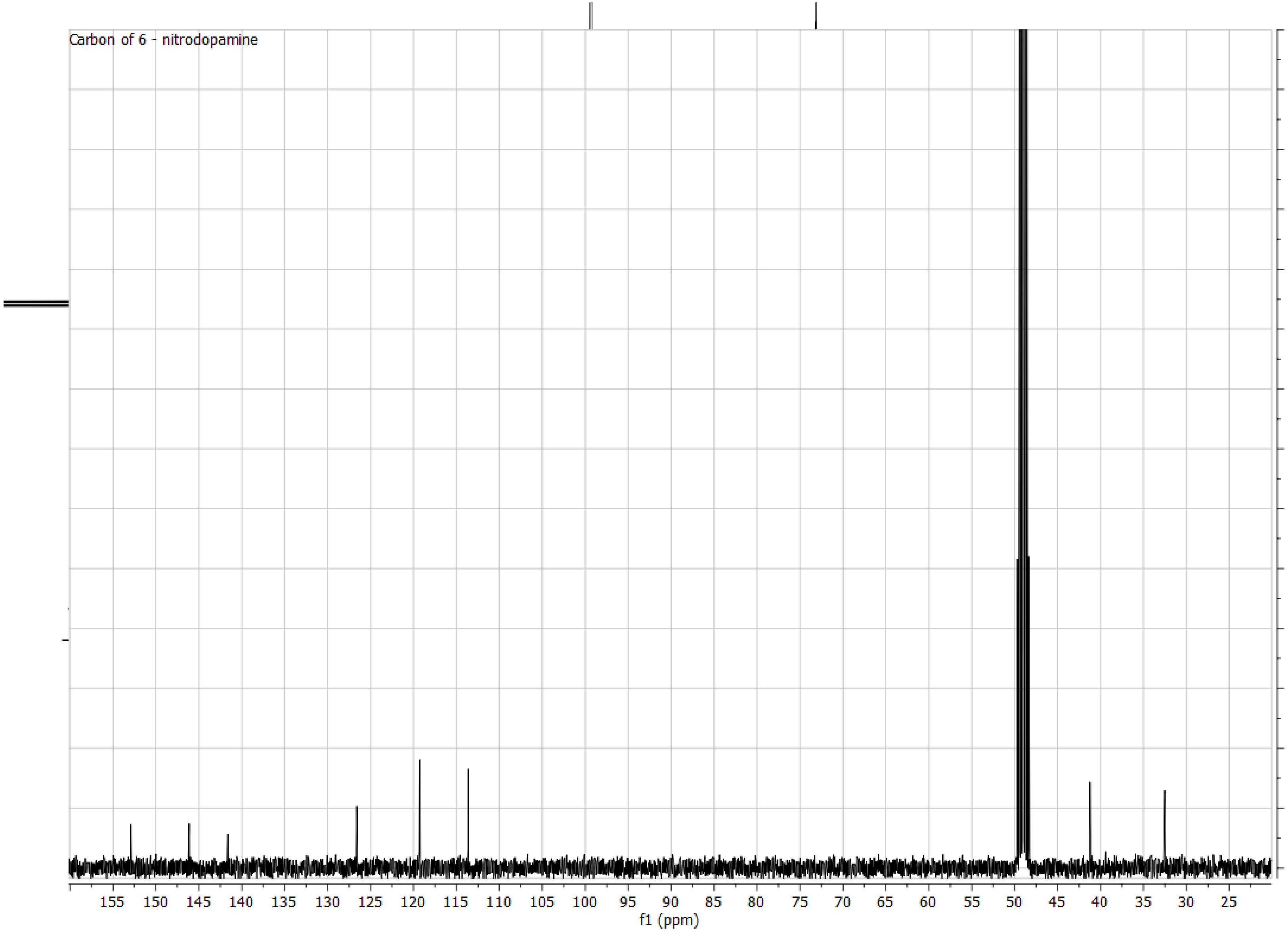

Figure S5 ^{13}C NMR spectra in CD_3OD of 6-ND.

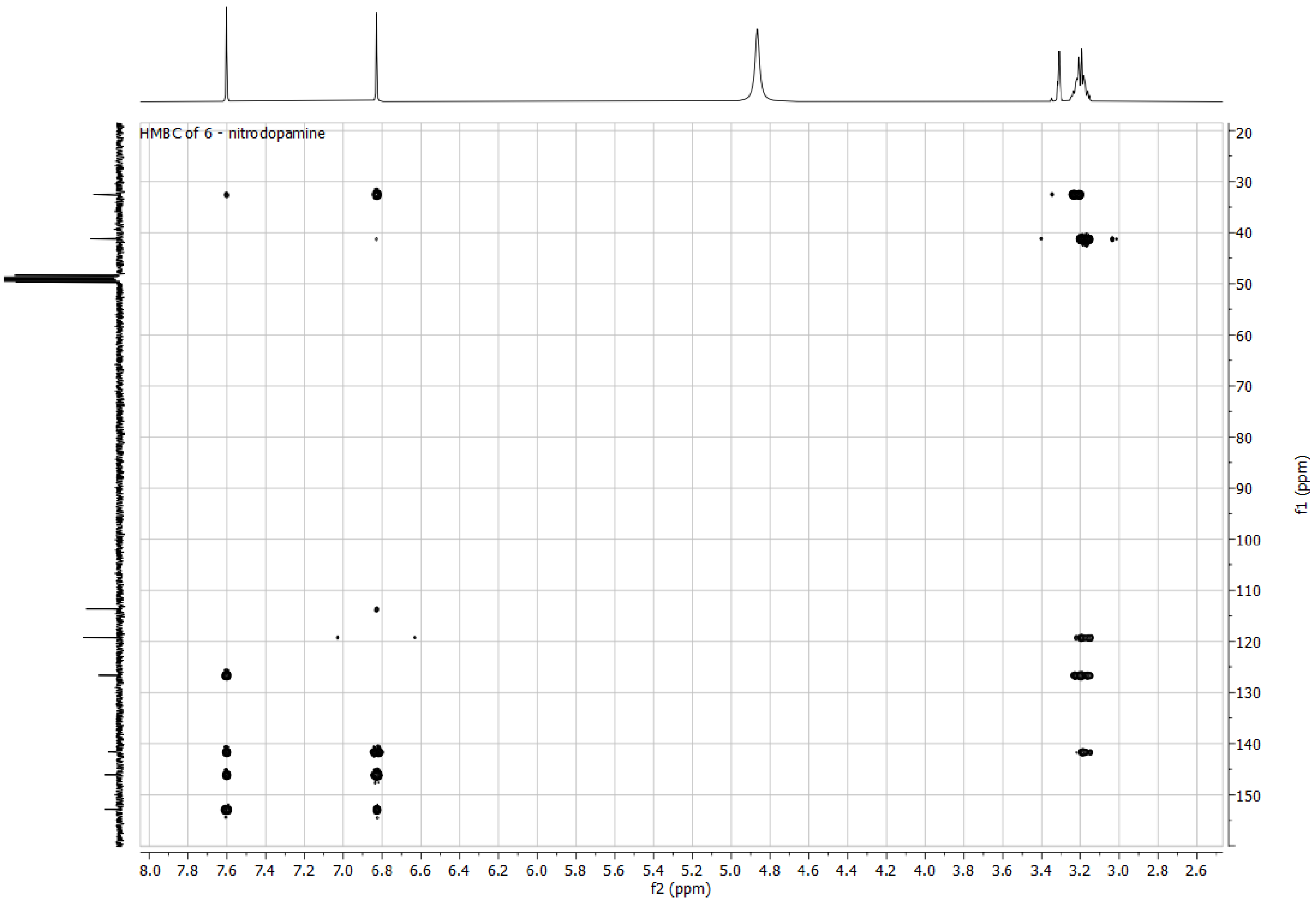

Figure S6 HMBC spectra in CD_3OD of 6-ND.

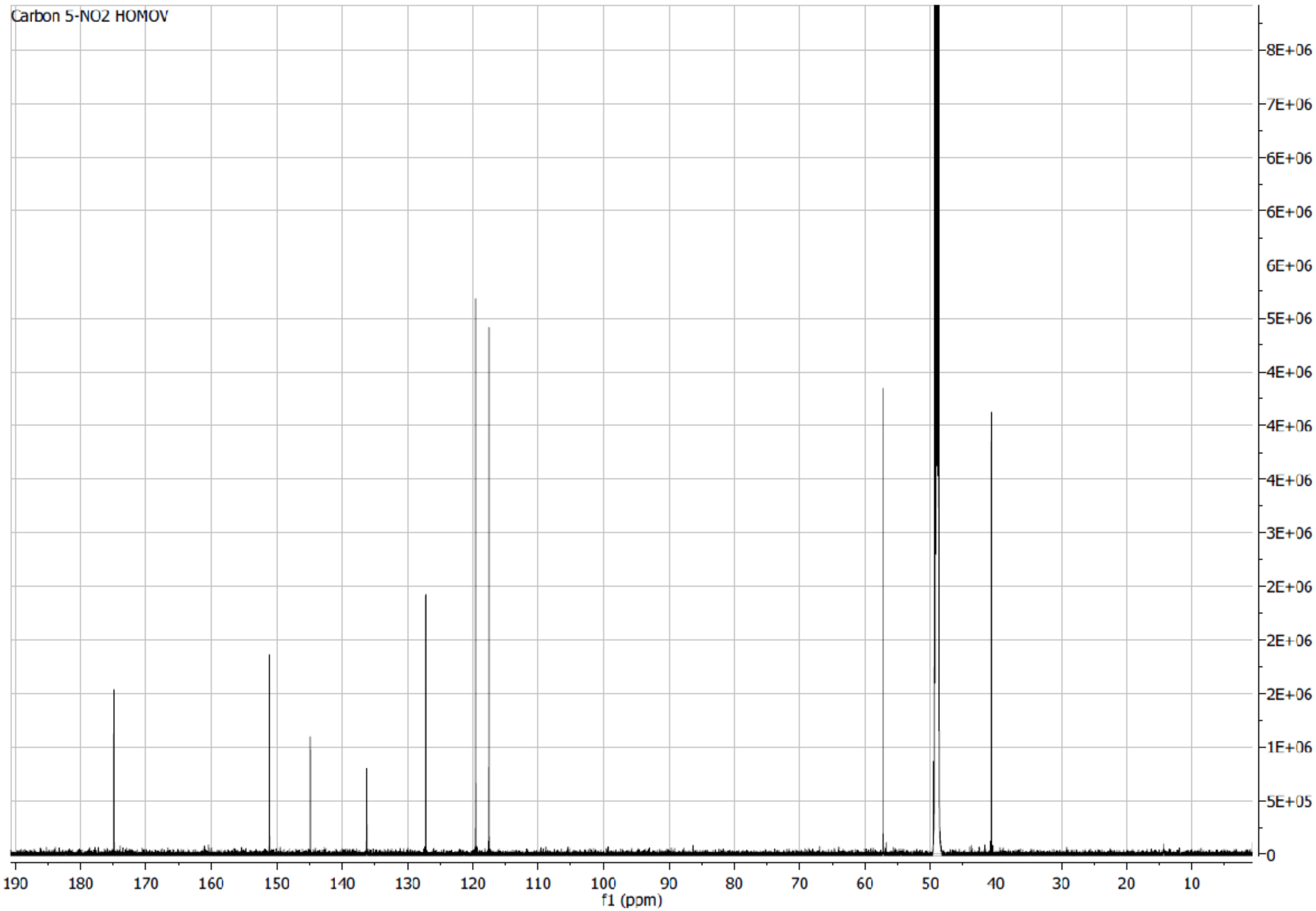

Figure S7 ^{13}C NMR spectra in CD_3OD of 5-NHVA.

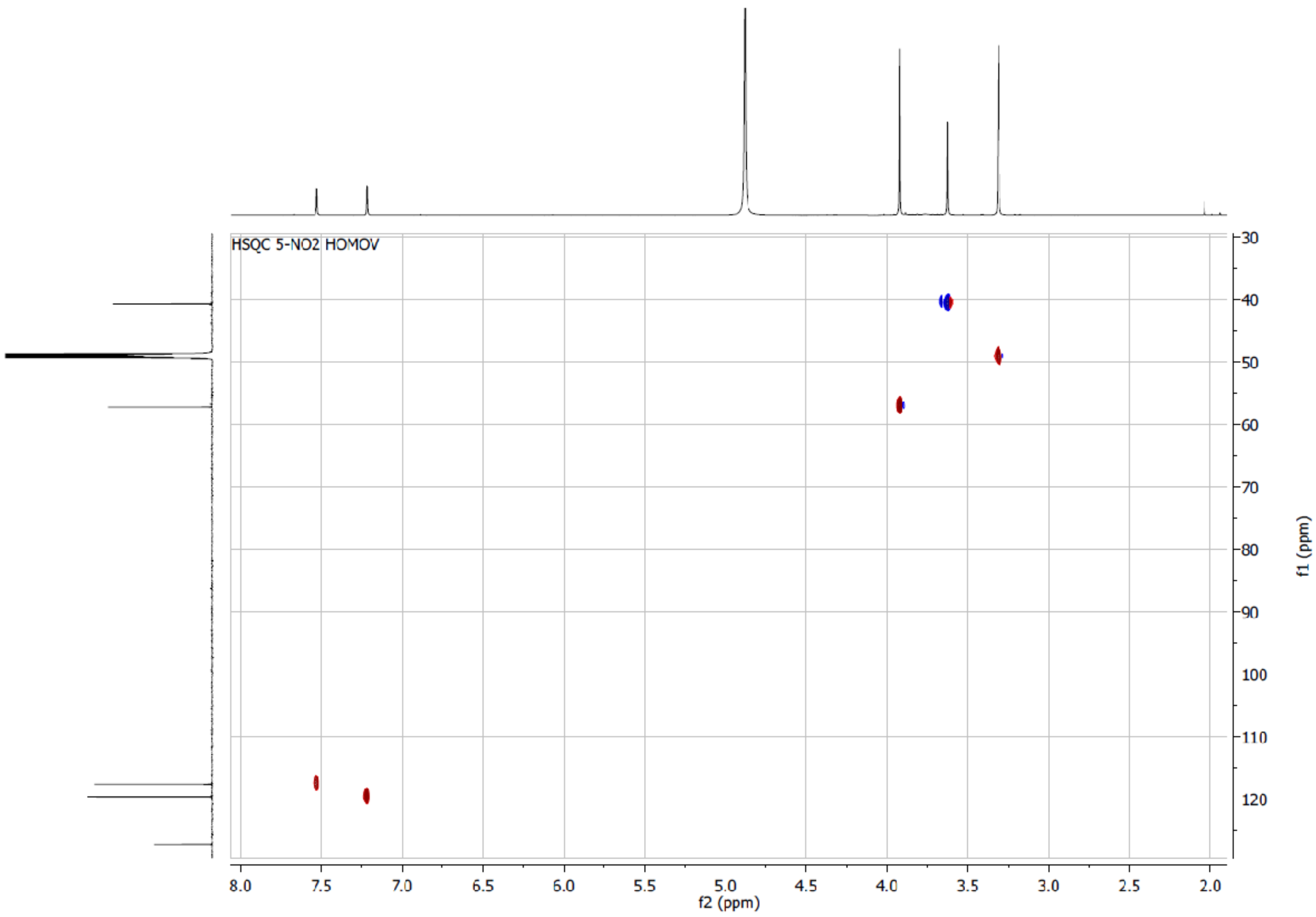

Figure S8 HSQC in CD_3OD of 5-NHVA.

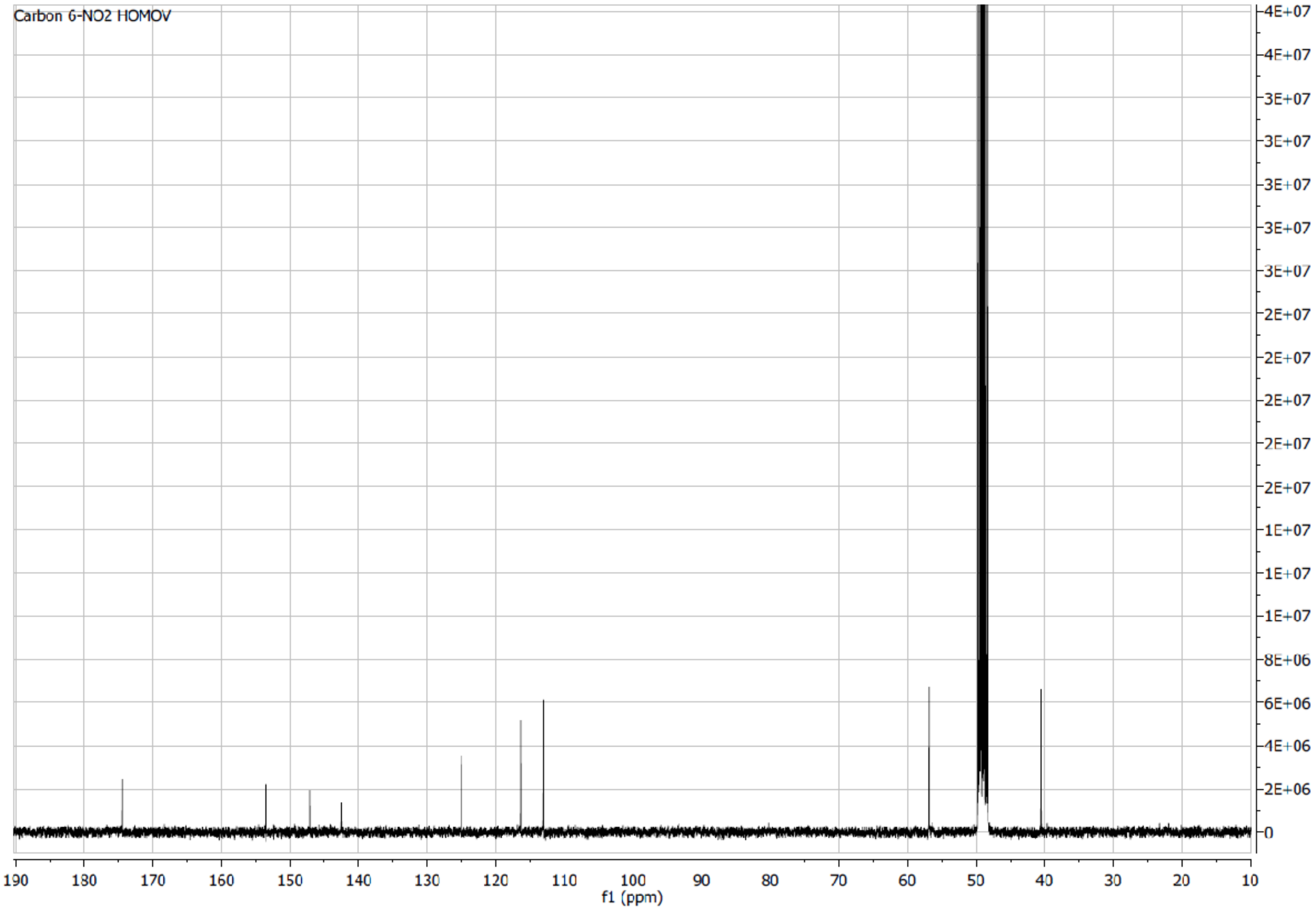

Figure S9 ^{13}C NMR spectra in CD_3OD of 6-NHVA

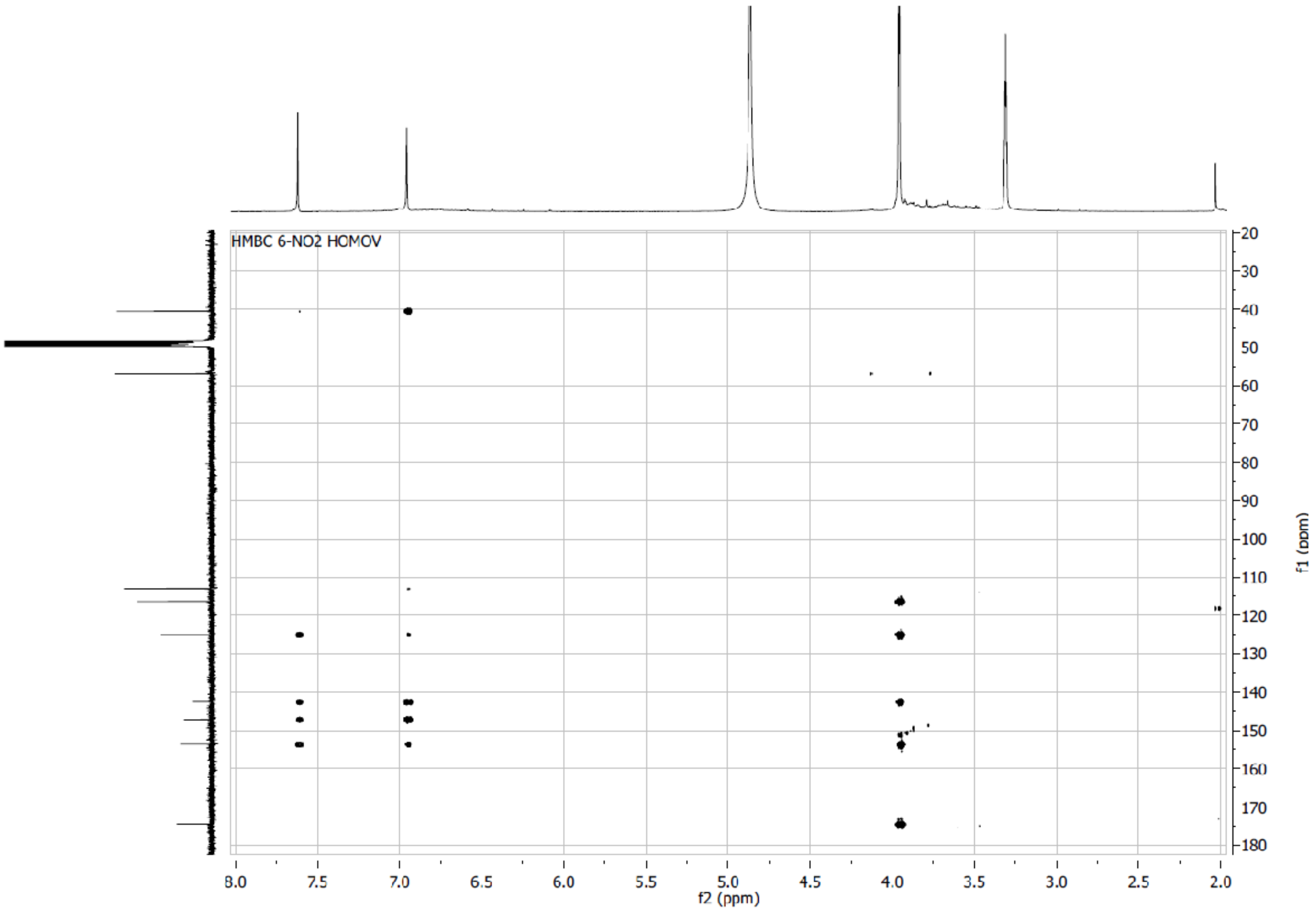

Figure S10 HMBC in CD_3OD of 6-NHVA.

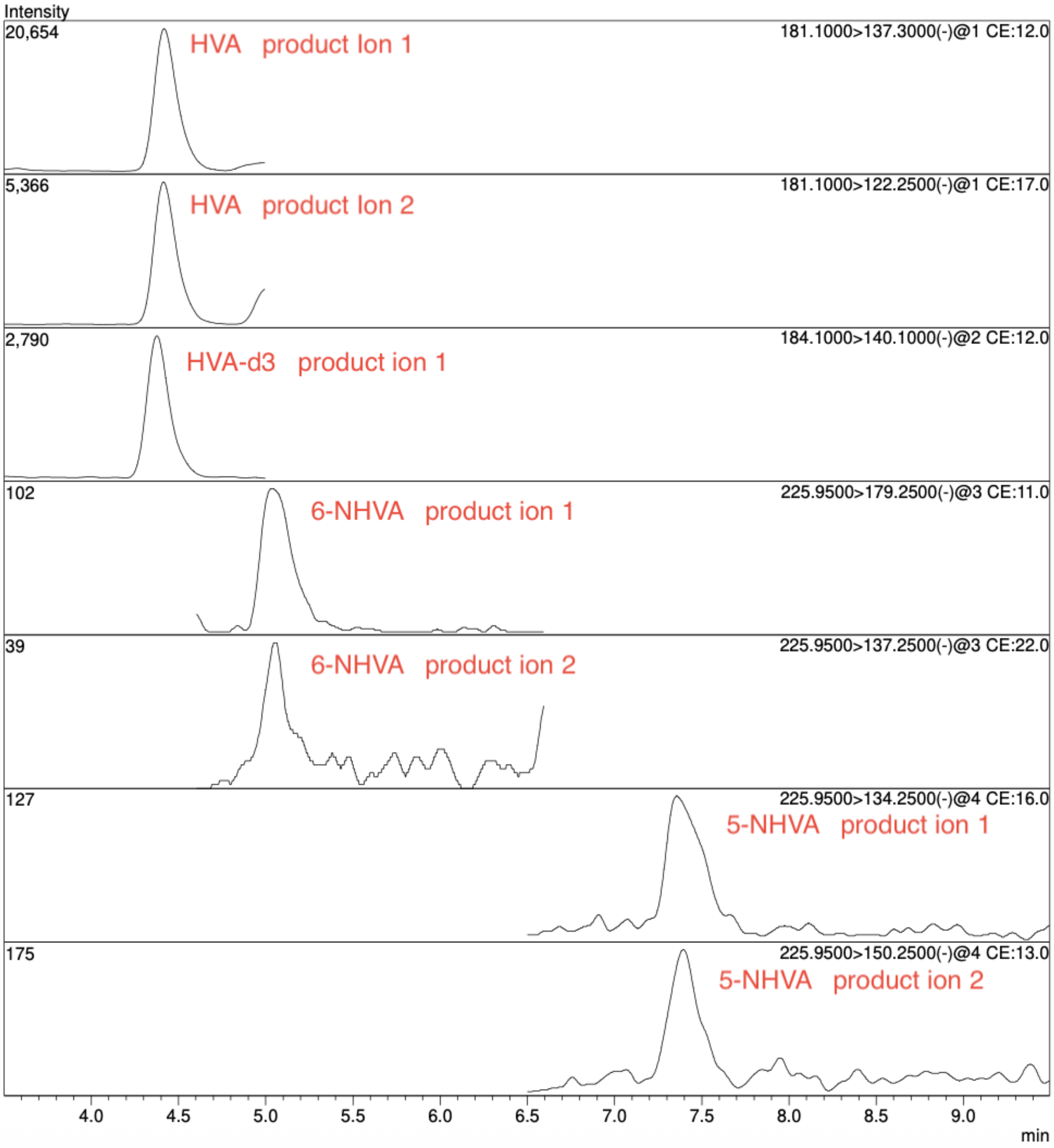

Figure S11. HVA, 6-NHVA and 5-NHVA identification by LC-MS/MS in human amniotic fluid sample. Each compound *Precursor Ion*, except the internal standard HVA-d3, had two fragments monitored (*Product Ion 1* and *Product Ion 2*). *Product ion 1* was used for quantification, while *Product Ion 2* was used for confirmation. *Precursor Ion* m/z is illustrated in each panel's top right legend, along with respective *Product Ion* m/z, and collision energies (CE). Negative Electron Spray Ionization was used in all cases. Refer to the method described in the main text for details.

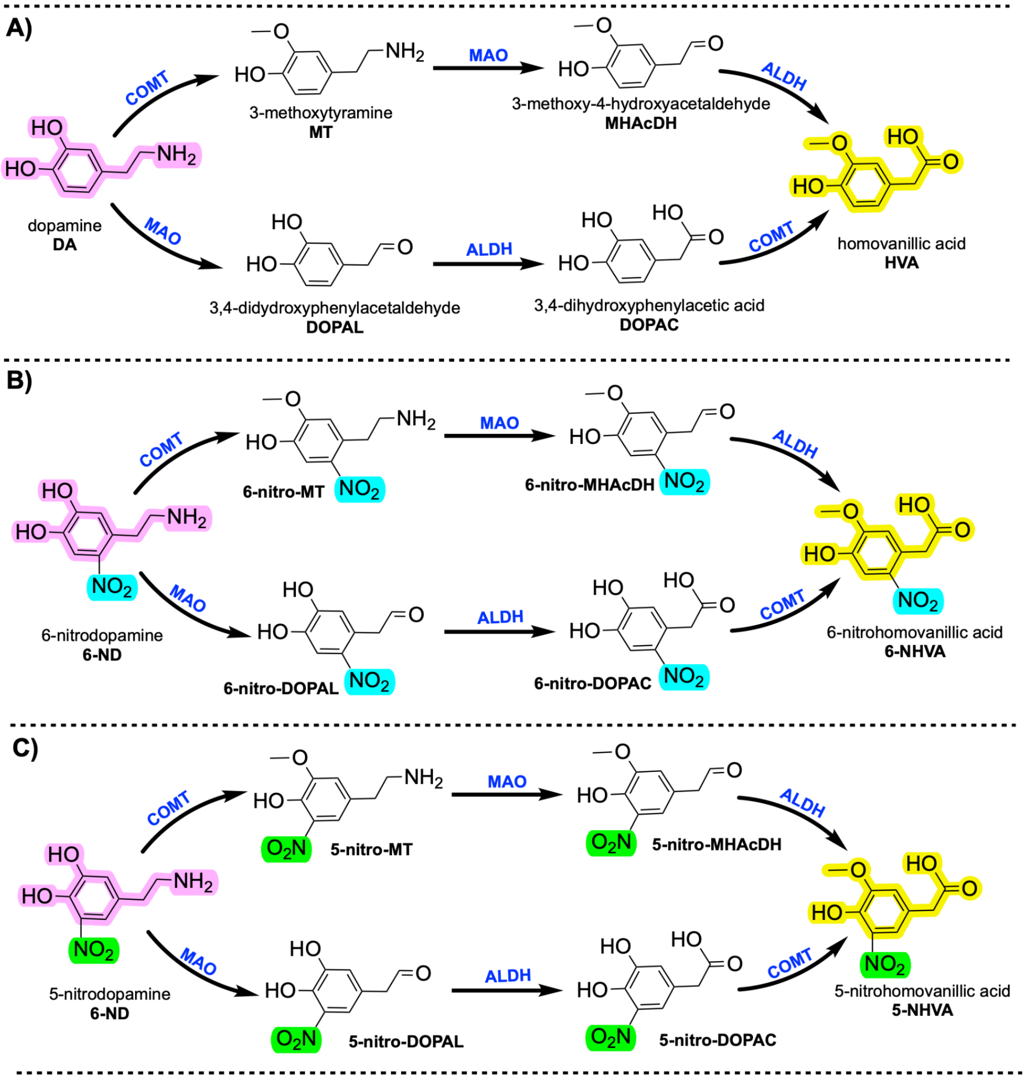

Scheme S1. Dopamine metabolism to homovanillic acid mediated by MAO, COMT, and ALDH enzymes **(panel A)**. Hypothetical 6-nitrodopamine **(panel B)** and 5-nitrodopamine (**panel C)** metabolism to 6-nitrohomovanillic acid and 5-nitrohomovanillic acid, respectively, under the assumption that the same enzymes known to metabolize dopamine can metabolize these nitroderivatives.
